# The Role of Lifestyle Nursing in Promoting Planetary Health: A Scoping Review Protocol

**DOI:** 10.1016/j.mex.2025.103659

**Published:** 2025-10-02

**Authors:** Anabela Coelho, Marta Sofia Catarino, Vanessa Cardoso Monteiro, Florbela Bia, Maria do Céu Marques, Mónica Couto Antunes

**Affiliations:** a Professor at Higher School of Nursing São João de Deus, Nursing Department. Universidade de Évora, Évora, Portugal; b Comprehensive Health Research Centre (CHRC), Universidade de Évora, 7000-811 Évora, Portugal; c Global Health and Tropical Medicine, Instituto de Higiene e Medicina Tropical, Universidade NOVA de Lisboa, 1099-085, Lisbon, Portugal; d PhD Student, Nursing Research, Center for Interdisciplinary Research in Health (CIIS), Universidade Católica Portuguesa, Lisbon,Porto, Portugal.; e Unidade Local de Saúde de Trás-os-Montes e Alto Douro, Unidade de Cuidados na Comunidade Vila Real I - Centro Académico Clínico de Trás-os-Montes e Alto Douro.

**Keywords:** Planetary Health, Environmental Sustainability, Nursing, Lifestyle Nursing, Health Promotion

## Abstract

This scoping review aims to explore and map the existing scientific evidence on how lifestyle nursing contributes to the promotion of planetary health. Lifestyle nursing encompasses interventions related to healthy behaviour change, including physical activity, nutrition, stress management, and sustainable practices. Planetary health, in turn, refers to the interconnectedness of human well-being and the health of natural systems.

The review will follow the Joanna Briggs Institute (JBI) methodology for scoping reviews and be reported according to the PRISMA-ScR guidelines. Relevant literature will be identified through comprehensive searches in databases such as CINAHL Ultimate, MEDLINE, Complementary Index, BASE (Bielefeld Academic Search Engine), Directory of Open Access Journals (DOAJ), Psychology and Behavioural Sciences Collection, and ScienceDirect, along with grey literature sources. Studies involving nurses or nursing interventions that address individual lifestyle, and environmental sustainability will be included. The results will provide a synthesis of current knowledge, highlight gaps in the literature, and inform future research and nursing practice related to sustainability and global health.

## Graphical abstract

**Figure:**
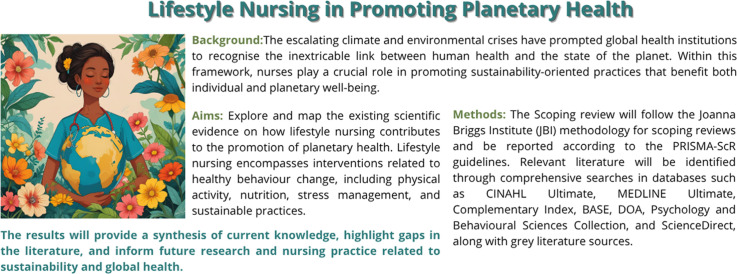

## Specifications table

TABLE 1: Specifications Table for the Scoping Review Protocol.

**Table:** 

**Subject area**	
**More specific subject area**	Sustainable Lifestyle Interventions in Nursing Practice for Planetary Health
**Name of your protocol**	The Role of Lifestyle Nursing in Promoting Planetary Health: A Scoping Review Protocol
**Reagents/tools**	N/A
**Experimental design**	N/A
**Trial registration**	N/A
**Ethics**	This study is a scoping review and does not involve the collection of primary data, experimentation involving human or animal subjects, or the use of personal data obtained from social media or other sources. It was based exclusively on published literature. Therefore, ethical approval and informed consent are not applicable.
**Value of the Protocol**	• Contributes to establishing lifestyle nursing as a central domain for promoting both individual and planetary health;
	• Supports interdisciplinary integration between nursing, lifestyle medicine, and sustainability;
	• Identifies knowledge gaps to inform research, practice, and policy;
	• Highlights the strategic role of nursing in advancing the Sustainable Development Goals (SDGs), particularly SDG 3 (Good Health and Well-being) and SDG 13 (Climate Action).

## Background

The escalating climate and environmental crises have prompted global health institutions to recognise the inextricable link between human health and the state of the planet, a concept now widely acknowledged as planetary health. Within this framework, nurses play a crucial role in promoting sustainability-oriented practices that benefit both individual and planetary well-being [[1], [2], [3]].

Lifestyle nursing, as an emerging field of practice, integrates behavioural change strategies, health promotion, and prevention with a growing emphasis on ecological responsibility. Nurses are uniquely positioned to influence personal health behaviours while simultaneously advocating for sustainable healthcare systems and community engagement [1,[4], [5], [6]].

Incorporating sustainability into nursing education, practice, and leadership has been increasingly documented in the literature. Recent studies emphasise the need to embed planetary health principles into nursing curricula and to empower nurses as agents of ecological change across clinical and community contexts [2,7,8]. Different frameworks have been proposed to operationalise sustainable actions in nursing care settings [3]. Nursing can incorporate the six pillars of a healthy lifestyle by educating patients on functional nutrition, promoting healthy sleep habits, encouraging conscious movement, fostering spirituality, supporting healthy relationships, and advocating for healthy environments, thus enhancing overall patient care and well-being [[9], [10]].

Despite growing recognition, the specific contributions of lifestyle nursing to the planetary health agenda remain underexplored and fragmented across this discipline. There is a need to synthesise and map the existing body of knowledge to inform future research, education, and practice. Scoping reviews are particularly suited to this task, as they enable a comprehensive mapping of the evidence regardless of methodological quality, offering insights into conceptual boundaries, interventions, and gaps in the literature [11].

Accordingly, this scoping review aims to explore and map the scientific evidence on how lifestyle nursing contributes to the promotion of planetary health.

A preliminary search conducted in February 2025 in the MEDLINE, Cochrane Database of Systematic Reviews, JBI Evidence Synthesis, and PROSPERO databases did not identify any type of literature review (published or in progress) on this specific topic.

## Description of protocol

### Review Question

What is the contribution of lifestyle nursing interventions to the promotion of planetary health?

Sub-questions:

1. What lifestyle nursing interventions contribute to planetary health?
2. In which community healthcare settings are these interventions applied?
3. What environmental outcomes - internal or external - are associated with these practices?
4. What facilitating factors and barriers are identified in the implementation of sustainable nursing practices?

## Materials and Methods

The scoping review will be conducted following the methodological guidelines provided by Joanna Briggs Institute [12]. Findings will be reported according to the Preferred Reporting Items for Systematic Reviews and Meta- Analyses extension for Scoping Reviews checklist [13].

The protocol is registered in the Open Science Framework (OSF) under the registration DOI: 10.17605/OSF.IO/Z5TPD. Initially developed in May 2025, the protocol underwent amendments in July 2025. The completion of the scoping review is projected for the end of November 2025.

### Eligibility Criteria- PCC Framework

Following the recommendations of JBI, the eligibility criteria consider the PCC mnemonic (P- Population; C- Concept; C- Context).

• Population

Inclusion criteria: Nursing professionals involved in health promotion, sustainable care, or lifestyle interventions.

• Concept

Lifestyle nursing refers to a field of nursing practice focused on promoting healthy behaviours, such as functional nutrition, regular physical activity, restorative sleep, stress management, and cultivating meaningful relationships and purpose. These interventions are aimed not only at individual health but also at contributing to planetary health through sustainable choices and practices [14,15].

• Context

This scoping review will prioritise healthcare or community settings, including primary care, public health, where planetary health or sustainable practices are addressed.

### Types of Sources

This scoping review will preferentially include peer-reviewed original studies (quantitative, qualitative, and mixed-methods), as well as reviews, concept papers, and theoretical frameworks published in English, Portuguese, or Spanish. A five-year time restriction will be applied. The search strategy will be adapted for each database and limited to studies published between January 2020 and May 2025.

Studies will be excluded if they are not related to nursing practice, if they focus exclusively on medical or environmental interventions without the involvement of nursing professionals, or if they consist of editorials or opinion pieces lacking empirical or theoretical contributions.

### Search Strategy

A three-step search strategy will be used in this review. An initial limited search of CINAHL completed via EBSCOhost and MEDLINE was undertaken to identify publications on the topic (Appendix 1). The keywords and phrases appearing in the titles and abstracts of the retrieved articles, as well as the indexing terms used to describe them, will be analysed. At the end of the first phase, we will conduct a word analysis to identify key terms, concepts, and recurring themes in the literature. This approach ensures that the review captures the full scope of relevant evidence and enhances the clarity and rigor of the methodology. Based on the results of this initial analysis, a second, comprehensive search will be conducted across the following databases: MEDLINE, CINAHL Ultimate, MedicLatina, Social Sciences Citation Index, BASE, Psychology and Behavioural Sciences Collection, Scopus and Web of Science, using the identified keywords and index terms. A detailed documentation will be carried out of all strategies used in each database, including search dates, terms, operators, and preliminary results. This ensures transparency and reproducibility, as recommended by the JBI. Finally, the reference lists of all included sources will be screened to identify any additional relevant literature.

The search strategy will combine three key concepts using the Boolean operator "AND": ("Planetary Health" OR "Environmental Sustainability"), (Nursing OR "Lifestyle Nursing"), and "Health Promotion" and limited to studies published in English, Portuguese, and Spanish.

### Data Collection and Analysis

• Selection of Studies

The records obtained from each information source will be transferred to Ryyan, where duplicate entries will be eliminated. To reduce potential bias, two reviewers will independently appraise the retrieved references in two sequential phases: (I) an initial appraisal of titles, abstracts, and descriptors to assess conformity with the predefined inclusion parameters; (II) a comprehensive evaluation of the full texts of all preliminarily eligible manuscripts. The appraisal procedure will employ a blinded assessment protocol, whereby each evaluator remains unaware of the other’s judgments throughout both the screening and in-depth review phases. In instances of discordance or ambiguity, a third evaluator will be engaged to facilitate consensus. The complete selection methodology will be illustrated using a PRISMA flow diagram - ScR, outlining the identification, screening, and inclusion stages culminating in the final sample of studies.

• Data Extraction

During the data extraction process, a descriptive assessment of each study will be conducted to collect information relevant to the review question and sub-questions. This includes the citation details, title, year, country, study type, study aim, lifestyle strategies, health promotion interventions, target population, and context (e.g., primary care, public health, community settings). Key findings, types of nursing interventions aligned with planetary health principles; settings of application (with particular attention to community-based contexts); internal and external environmental outcomes of interventions; and barriers and facilitators to the implementation of sustainable nursing practices will also be extracted. Findings will be extracted from a specific instrument developed by the research team (Appendix 2). The draft data extraction tool will be modified and revised as necessary during data extraction from each included evidence source.

• Quality Appraisal

Following the methodological framework for scoping reviews, no formal critical appraisal of the included studies will be performed, as the primary objective is to map the existing evidence regardless of methodological quality. However, key characteristics of each study will be charted to provide a comprehensive overview of the available literature.

• Strategy for Data Synthesis

Data synthesis will follow a descriptive-analytical approach, in accordance with the JBI methodology for scoping reviews. The data extracted from the included studies will be organized in tabular format and subsequently presented in the “Results” section through a narrative description that provides context and facilitates interpretation of the findings.

Additionally, visual representations, in the form of tables, graphs, and/or figures, may be developed to facilitate cross-study comparison and enhance the accessibility and clarity of the results.

All researchers will actively participate in the synthesis process to ensure clarity, consistency, and methodological rigour in data presentation. This approach will enable a comprehensive mapping of the scope, nature, and characteristics of the available evidence, contributing to a deeper understanding of the role of lifestyle nursing in promoting planetary health and advancing the Sustainable Development Goals.

• Data Availability Statement

All extracted data underlying the findings of this scoping review will be made publicly available without restriction upon publication of the final article. The data, including detailed characteristics of the included studies and extracted outcomes as per Appendix 2 instrument, will be deposited in spreadsheet format within the public Open Science Framework (OSF) repository, linked to this protocol's project (DOI: 10.17605/OSF.IO/Z5TPD), to ensure transparency and reproducibility."

### Protocol validation

The protocol was validated through consultation with experts ensuring methodological rigour and the relevance of the study design. Experts with a minimum of 10 years of professional or academic experience in their respective fields were included, as well as those with relevant publications or recognised contributions in the area.

A total of 5 experts participated: all in nursing, with experience in evidence synthesis/methodology, and two in planetary health. The process followed two rounds: in the first phase, the experts individually reviewed the protocol and submitted written comments; in the second phase, a consensus meeting (online group discussion) was held, which allowed the refinement and synthesis of the suggestions presented. Additionally, the protocol was internally reviewed by the research team and externally validated through its registration on the Open Science Framework platform, ensuring transparency and accountability throughout the entire review process.

The progressive development and refinement of the protocol ensured its alignment with the PRISMA-ScR and Joanna Briggs Institute (JBI) guidelines.

During the data extraction phase, a pilot test of the data extraction form will be conducted on a sample of studies to verify whether it consistently captures the necessary information.

Any substantial changes made during the review process will be adequately documented and justified in the final report, safeguarding the methodological integrity and reproducibility of the study and providing a foundation for integrating planetary health competencies into nursing education and leadership.

## Limitations

None

## CRediT author statement

Conceptualization: F.B., M.C., M.A., A.C. and V.M; Methodology: A.C.; F.B.; M.C., M.M., M.A. and V.M.; Resources: F.B., M.C., M.M. and M.A.; Writing—original draft: F.B.; M.C; M.A. and V.M.; Writing—review and editing: A.C.; Supervision: A.C. All authors have reviewed and approved the final text of the manuscript.

## Declaration of competing interests

The authors declare that they have no known competing financial interests or personal relationships that could have appeared to influence the work reported in this paper.

## Figures and Tables

**Table 1:** Search strategy for MEDLINE and CINAHL. The search was conducted on May 2025.

Search	Query	Record Retrieved
**CINAHL**	("Planetary Health" or "Environmental Sustainability") AND (Nursing or "Lifestyle Nursing") AND "Health Promotion"	328
**MEDLINE**	("Planetary Health" or "Environmental Sustainability") AND (Nursing or "Lifestyle Nursing") AND "Health Promotion"	315

**Table 2:** Data extraction instrument.

***Title:****The Role of Lifestyle Nursing in Promoting Planetary Health: A Scoping Review Protocol*	
***Review aim:****To explore and map the scientific evidence on how lifestyle nursing contributes to the promotion of planetary health.*	
***Review question/s:*** *What is the contribution of lifestyle nursing interventions to the promotion of planetary health?* ***Sub-questions:*** *1. What lifestyle nursing interventions contribute to planetary health?* *2. In which community healthcare settings are these interventions applied?* *3. What environmental outcomes - internal or external - are associated with these practices?* *4. What facilitating factors and barriers are identified in the implementation of sustainable nursing practices?*	
***Inclusion/Exclusion Criteria***	
*Population*	
*Concept*	
*Context*	
*Types of evidence source*	
***Evidence source Details and Characteristics***	
Citation details	
*Authors*	
*Year*	
*Country*	
Study aims/ goals	
Participants	
Key findings	
Settings of application (with particular attention to community-based contexts	
*Lifestyle nursing interventions contribute to planetary health*	
Types of nursing interventions aligned with planetary health principles	
*Environmental outcomes (internal or external) associated*	
*Facilitating factors identified in the implementation of sustainable nursing practices*	
*Barriers identified in the implementation of sustainable nursing practices*	
*Comments*	

## Data Availability

Data will be made available on request.
